# Electrical and Photodetector Characteristics of Schottky Structures Interlaid with P(EHA) and P(EHA-*co*-AA) Functional Polymers by the iCVD Method

**DOI:** 10.1021/acsomega.3c04935

**Published:** 2023-11-28

**Authors:** Selçuk Demirezen, Murat Ulusoy, Haziret Durmuş, Halit Cavusoglu, Kurtuluş Yılmaz, Şemsettin Altındal

**Affiliations:** † Sabuncuoğlu Şerefeddin Vocational School of Health Services, Amasya University, 05100 Amasya, Turkey; ‡ Department of Physics, Gazi University, 06500 Ankara, Turkey; § Department of Physics, Faculty of Science, Selçuk University, Selçuklu, 42130 Konya, Turkey; ∥ Chemical Engineering Department, Konya Technical University, 42030 Konya, Turkey

## Abstract

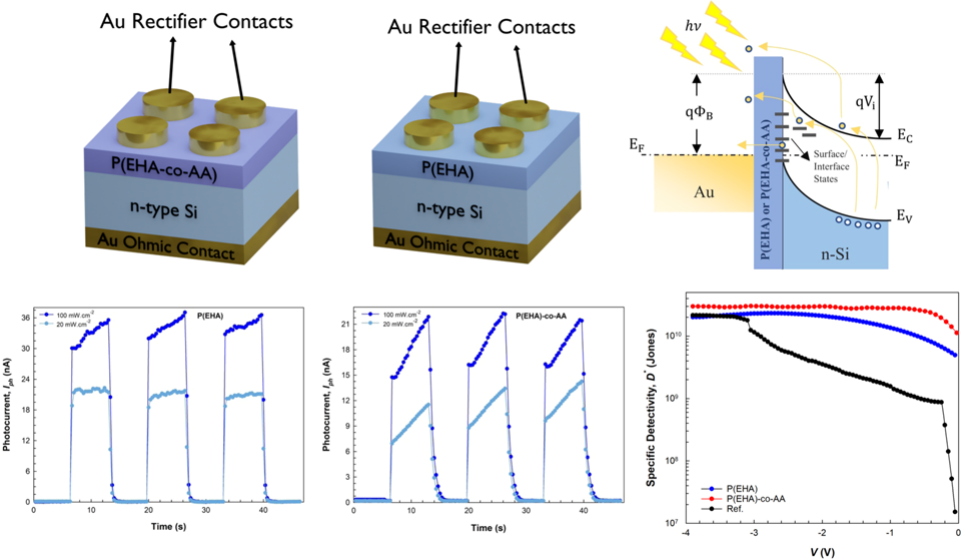

In this study, poly(2-ethylhexyl acrylate) (PEHA) homopolymer and its copolymer combined with acrylic acid P(EHA-*co*-AA) were employed as interfaces in two separate Schottky structures. First, both interfaces were grown by initiated chemical vapor deposition (iCVD), which provides much better deposition control and homogeneous coating compared to solution-phase methods. In addition to this advantageous method, the effects of two different polymers, one of which is better able to adhere to the crystal surface on which it is formed than the other, on the optoelectronic properties have been studied. Then, their current–voltage (*I*–*V*) and capacitance/conductance–voltage (*C*/(*G*/ω)–*V*) characteristics were investigated both in the dark and under illumination. The basic electrical parameters and the illumination-induced profile of the surface state (*N*_ss_) were probed by *I*–*V* and *C*–*V* measurements for two samples. A decrease in the barrier height (BH) and, consequently, a significant increase in the photocurrent were observed under illumination. Striking changes in series resistance (*R*_s_) values are also highlighted. The photocapacitance and conductance characteristics indicated that the structures could be considered not only as photodiodes but also as photocapacitors. Moreover, the voltage-dependent changes of some photodetector parameters, such as responsivity (*R*), sensitivity (*S*), and specific detectivity (D*), along with the transient photocurrent characteristics, are discussed for both structures. Therefore, we can say that the strong changes in these parameters, which figure the merit of photodiode and photodetector applications, depending on the voltage and under illumination, prove that it is a study carried out in accordance with the purpose and so they can be used in electronic and optoelectronic applications.

## 1 Introduction

The photovoltaic effect allows solar cells (SCs) and photodetectors (PDs) to directly convert the energy from sunlight into direct current (dc) electricity. To achieve this conversion, a material is needed that can absorb sunlight and allow these high-energy electrons to move through an external circuit. The optical parameters and charge transport mechanisms of these devices depend on various parameters, such as fabrication preparations, surface contamination, surface/interface state intensity distributions (*N*_ss_), the form of the barrier height (BH), series resistance (*R*_s_), and executed bias voltage. Traditionally, the basic scientific/technical problems of these devices are also relevant to the increase in efficiency and to reducing energy losses and costs. Therefore, for inorganic silicon-based electronic structures, many studies have been carried out on organic silicon-based structures, which have been a strong alternative in many respects for a while. Herewith, polymers can be shown to be one of the most susceptible candidates for organic structures interlaid at the M–S interface. They are generally separated from inorganic structures by their commercial availability, controllable electrical and mechanical properties, and ease of fabrication in device electronics and optoelectronics studies.^1−3^ Moreover, the structural nature of organic polymers allows for significant developments in flexible electronics applications, such as curved imaging and wearable device technologies.^4−9^

Among the flexible functional polymers that can physically adhere strongly to the surface without altering the morphological properties of the surface in thin-film coating applications, there are remarkable advantages in terms of both functional chemical diversity and applicability to different substrates.^10,11^ Thanks to its low glass-transition temperature and its ability to form homogeneous films, poly(2-ethylhexyl acrylate) (PEHA) is one of the functional polymers that stand out among self-adhesive polymers.^12−14^ Therefore, such polymers have applications in various fields, such as the adhesive industry, medical applications, biomaterials, and sensor applications.^15^ In this study, it was employed in the Schottky structure as an interface. In addition, the copolymer combined with acrylic acid, which was reported to increase the adhesion ability,^16,17^ was also employed as a second Schottky structure interface. The use of polymers as insulating interfaces in Schottky structures has a considerably long history.^18^ They are widely used in organic SCs and PDs, mainly due to their properties, as mentioned above, and also their high transparency.^19−26^ Today, it can be said that it is often preferred in optoelectronic applications, which are gradually developing. Polymers have been used as interfaces in such studies in their pristine form or by doping with various filling nanostructures (metals, metal oxides, graphene, GO, etc.) and even cation- and anion-bonded materials.^27,28^

The physicochemical interactions underlying the preference of polymers and/or doped polymers as active medium/interface in photovoltaic devices are basically based on the photogeneration of e^–^–h^+^ couples depending on the energy irradiated onto the surface of the device.^29,30^ In addition, there are studies that include polymer and copolymer compositions in such device applications, albeit in very limited numbers.^31,32^ Regardless of the layer deposition method or interface material chosen, the main purpose of a photovoltaic structure is to achieve reasonable photocurrent values as a function of the intensity and/or wavelength of the incident light.^33^ The production and development of sensors sensitive to visible light and/or its near-neighbor radiation (NIR or UV) have become a necessity for autonomous systems and devices that are making their presence felt in many areas of our lives.

In line with this necessity, in the present study, poly(2-ethylhexyl acrylate) (PEHA) homopolymer and its copolymer combined with acrylic acid P(EHA-*co*-AA) were employed as interfaces in two separate Schottky structures. Both interfaces were fabricated by the initiated chemical vapor deposition (iCVD) method, which provides much better deposition control and homogeneous coating compared to solution-phase methods. The current–voltage (*I*–*V*) and capacitance/conductance–voltage (*C*/(*G*/ω)–*V*) measurements of the fabricated structures were executed at certain voltage ranges in the dark and under illumination (100 mW cm^–2^). The basic electrical diode parameters, the energy- and voltage-dependent distributions of the surface state intensities (*N*_ss_), and some photodetector parameters, such as responsivity (*R*), sensitivity (*S*), and detectivity (D*), have been studied. A decrease in the barrier height and, consequently, a significant increase in the photocurrent were observed under illumination. At the same time, peaks in the *S* and D* parameters, especially around −0.5 V, indicate that the photodetector properties of the formed structures are strong. Therefore, we can say that the strong changes in these parameters, which figure the merit of a photodetector, depending on the voltage under illumination, prove that this is a study carried out in accordance with the purpose.

In this study, we aimed to investigate the effects of two different polymers (homopolymer and copolymer), one of which can adhere better to the crystal surface on which it is formed than the other, on the surface states that were also investigated. Perhaps, the different intensities of the *N*_ss_ distributions according to the energy range are due to the differences in these adhesion properties of the polymers and/or to the effects of the atoms/charges localized at the polymer/semiconductor interface. However, there are almost no studies in the literature on whether the adhesion properties of polymers have an effect on the surface states. Therefore, specific studies to be conducted in this area will allow for more satisfactory results to be obtained at this point.

## 2 Experimental Section

### 2.1 Materials

Substrates, including silicon wafers (∼300 μm thickness, ⟨100⟩, n-type, and 1–10 Ω·cm), were employed in the study. The monomers utilized were 2-ethylhexyl acrylate (EHA; Aldrich, 98%) and acrylic acid (AA; Aldrich, 99%), while the initiator was di-*tert* butyl peroxide (TBPO; Aldrich, 98%). All precursors were utilized in their as-received form without any modification or purification.

### 2.2 iCVD of P(EHA) and P(EHA-*co*-AA) Films and Fabrication of the Structures

**Figure 1:**
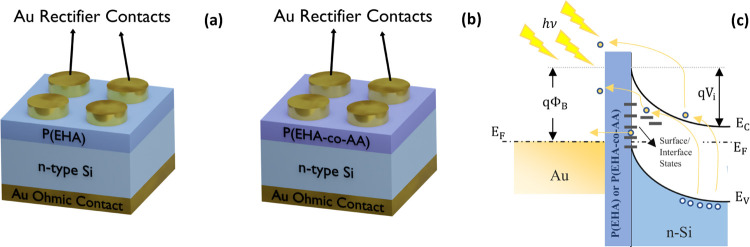
Pictorially fabricated structures: (a) interlaid with the P(EHA), (b) interlaid with the P(EHA-*co*-AA), and (c) band diagram with some electron conduction mechanisms.

After the substrate was chemically cleaned, the backside was thermally coated with high-purity (99.999%) gold (Au) at 10^–6^ Torr pressure for ohmic contact with a thickness of 150 nm in order to fabricate the structures interlaid with the polymer and copolymer in the first step. The deposition was then performed on 1 cm × 1 cm substrates using a custom-built cylindrical stainless steel vacuum chamber measuring 50 cm in diameter and 75 cm in length. Further details of the system can be found in the cited study.^14^ A vacuum was attained by using a rotary vane vacuum pump (2XZ-15C, EVP). The substrates were inserted into the reactor, and the temperature of the samples was anchored at 25 °C using recirculating chiller water (Lab. Companion RW-0525G, South Korea). The necessary heat energy for reaction initiation was harnessed by a nichrome (Ni–Cr 80/20 wt %, 0.3 mm diameter) filament array, placed 2.5 cm above the substrate surface, and the filament temperature was maintained consistently at 240 °C during the reaction. The temperature of the reactor wall was held at a constant 50 °C. A capacitance-type pressure sensor (MKS, Baratron, 1 Torr) was utilized to measure the chamber pressure, which was maintained at a continuous pressure of 600 mTorr throughout all depositions. The monomers, EHA and AA, and the initiator, TBPO, were placed in stainless jars for vaporization into the reactor. The temperature of the jars was set at 70 °C, 40 °C, and room temperature for EHA, AA, and TBPO, respectively. The initiator/total monomer ratio (I/MT) was kept consistent at 1/1 for all depositions. For homopolymer P(EHA) deposition, EHA and TBPO vapors were introduced into the chamber at a pressure ratio of 300/300. In order to deposit the copolymer, a constant EHA/AA pressure ratio of 100/200 was employed. After the deposit processes, ∼1 mm diameter Schottky dot contact formation was carried out on the thin films using high-purity Au (0.999). The pictorially fabricated structures are shown in Figure 1a–c, along with a band diagram, illustrating some electron conduction mechanisms involved.

## 3 Results and Discussion

### 3.1 Film Surface Morphology

**Figure 2:**
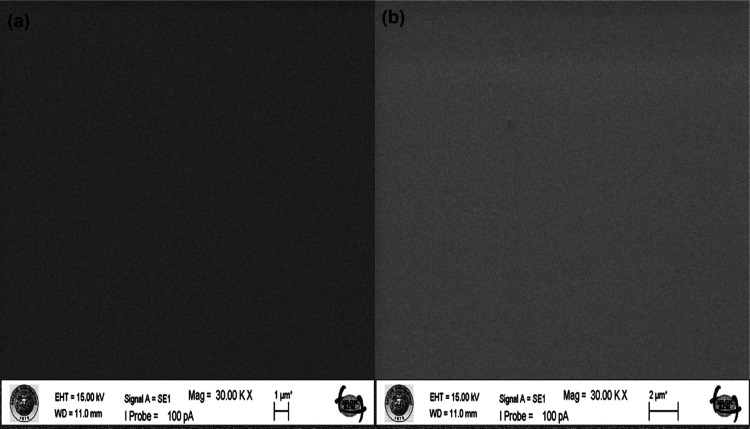
SEM images of the P(EHA) (a) and P(EHA-*co*-AA) films (b) deposited from iCVD.

Figure 2 shows the SEM images of the P(EHA) and P(EHA-*co*-AA) films that were deposited by iCVD at the same filament temperatures when the M/I ratio was 1. The films deposited at the 240 °C filament temperature are smooth and featureless, which is true of almost all PEHA films deposited under various conditions. Similar findings have been reported by other researchers.^11^

In this study, the highest occupied molecular orbital (HOMO) and lowest unoccupied molecular orbital (LUMO) energies and energy gap between them were calculated for P(EHA) and P(EHA-*co*-AA) functional polymers. Especially, the HOMO–LUMO band gap is a significant parameter for the determination of molecular electrical transport. In computational quantum chemistry, the energy of HOMO stands for the ionization potential and the energy of LUMO stands for the electron affinity.^34^ Moreover, it can be said that a molecule with a high HOMO–LUMO energy gap has a high kinetic stability and low chemical reactivity. The calculated LUMO energies of the polymers are −0.28 eV for P(EHA) and −0.32 eV for P(EHA-*co*-AA). The HOMO energies of the polymers were calculated as −6.54 and −6.37 eV, respectively. The energy gaps were calculated as 6.26 and 6.05 eV, respectively. This result indicates that the band gap for the copolymer is smaller than the main polymer, resulting in lower molecular stability and greater chemical reactivity. Scientific studies show that increased chemical reactivity can have positive effects on the physical and chemical properties of a molecule.^35^

### 3.2 Film Structure

**Figure 3:**
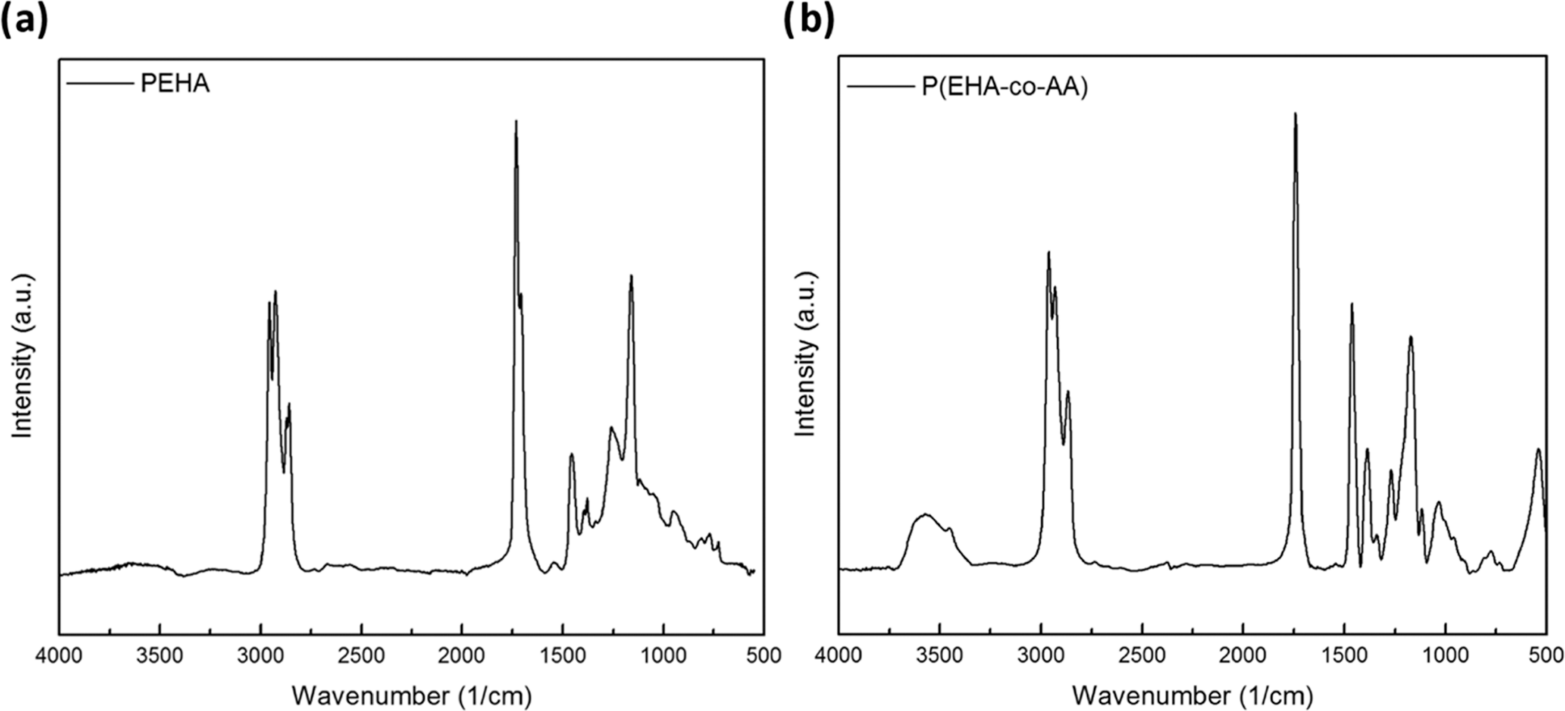
FTIR spectra of iCVD polymers P(EHA) with the spectra of corresponding monomers EHA (a) and FTIR spectra of iCVD P(EHA-AA) copolymers (b).

Figure 3a illustrates the FTIR spectra of P(EHA), both of which were deposited via iCVD, in addition to the spectra of their corresponding monomers. The P(EHA) spectrum exhibits four primary vibrational modes: a robust C=O stretching peak at 1733 cm^–1^, C–O stretching peaks in the range of 1000–1250 cm^–1^, bending vibrations of −CH_2_ or −CH_3_ between 1380 and 1461 cm^–1^, and stretching vibrations of C–H at 2860, 2929, and 2959 cm^–1^. P(EHA-AA) copolymer was synthesized, and their FTIR spectrum is displayed in Figure 1b. Copolymer spectrum displayed characteristic peaks from both EHA and AA monomers. The FTIR spectrum of P(AA) exhibits a distinct carbonyl stretching peak at 1707 cm^–1^, as well as a characteristic broad O–H stretching vibration peak centered at 3056 cm^–1^. This indicates the inclusion of an acrylic acid unit in the copolymer structure.

### 3.3 *I*–*V* Characteristics

**Figure 4:**
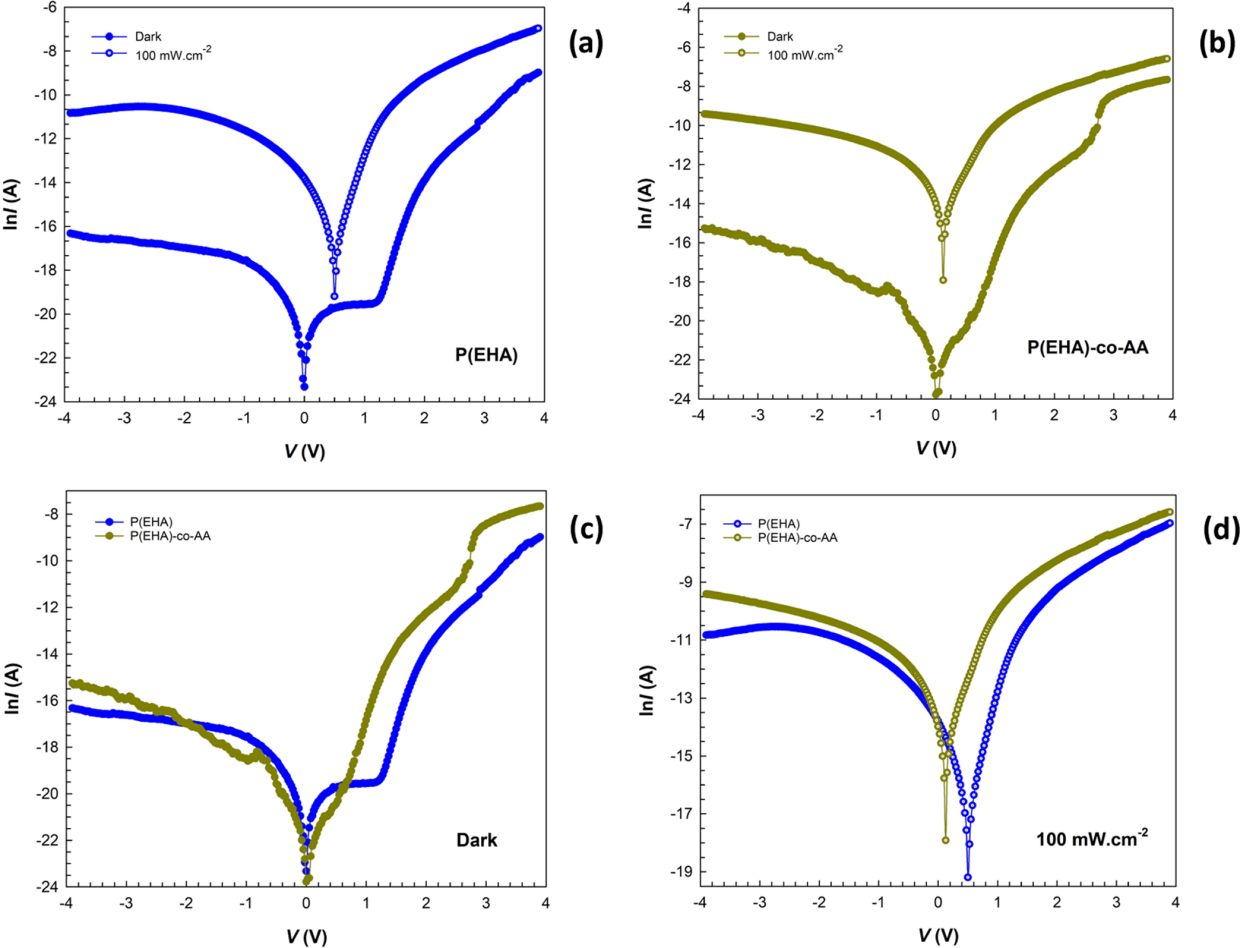
ln* I* vs *V* plots of the structures interlaid with the (a) P(EHA) in the dark and under illumination, (b) P(EHA-*co*-AA) in the dark and under illumination, (c) P(EHA) and P(EHA-*co*-AA) in the dark, and (d) P(EHA) and P(EHA-*co*-AA) under illumination.

Investigation of *I*–*V* and *C*/*G*–*V* measurements as a function of illumination yields exciting findings about the device’s electrical characteristics. MS structures with a thin insulator-oxide or polymer interface are particularly sensitive to illumination. If the energy of the absorbed photon is greater than the band gap energy of the semiconductor material, electrons will migrate to the rectifier metal either by being excited to the conduction band and crossing the interfacial barrier from there or tunneling via interface states/trap levels. The forward and reverse bias *I*–*V* characteristics of structures with different interlayers P(EHA) and P(EHA-*co*-AA) were measured in the dark and under 100 mW cm^–2^ illumination and given in Figure 4a–d, respectively.

**Figure 5:**
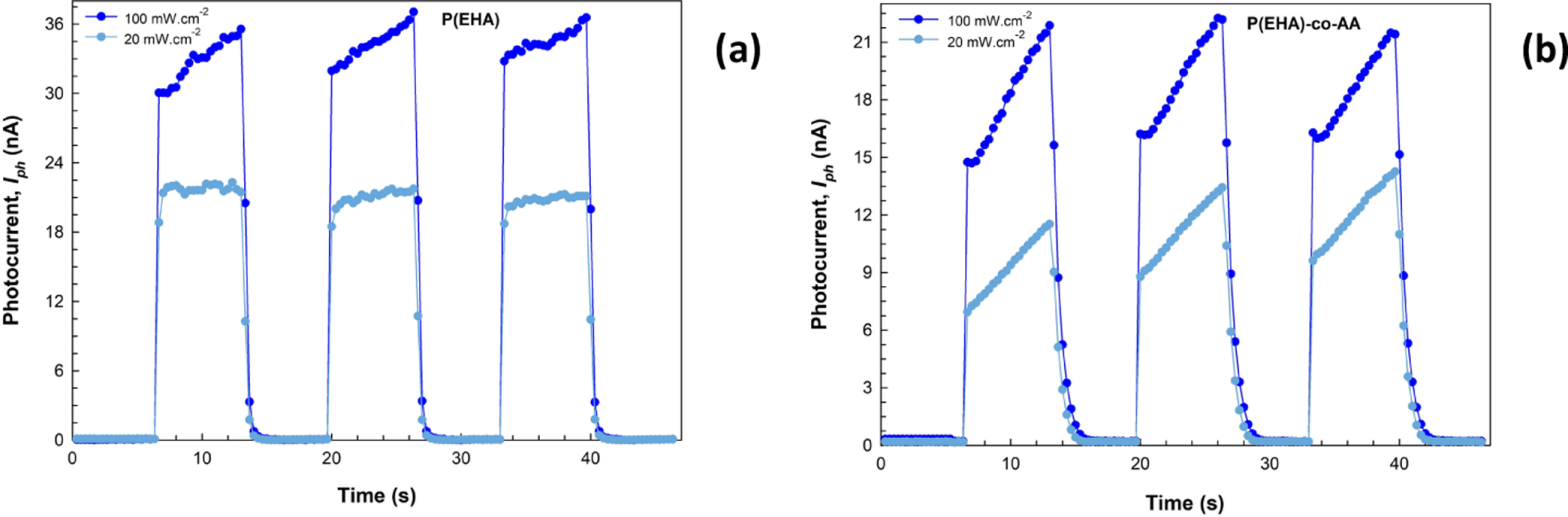
Transient photocurrent vs time plots of the structures interlaid with (a) P(EHA) and (b) P(EHA-*co*-AA).

The photocurrent values (*I*_ph_) rise as the intensity of illumination increases, particularly in the reverse bias region due to the photogenerated e^–^–h^+^ pairs under illumination, as shown in the figures. Put differently, when exposed to light, a significant number of these pairs are generated, leading to the manifestation of photoconductive characteristics. This behavior can be explained by the fact that after absorbing enough energy (*hc*/*q*λ ≥ *E*_g_), the valence band electrons move to the conduction band. The recombination of e^–^–h^+^ pairs can be hindered by a strong electric field in the reverse bias region, whereas it is unaffected by a low electric field in the forward bias region.^36−38^ Begin with, by studying the on/off time characteristics of zero-bias photocurrents, we can examine the photoresponse properties of the structures. The plots of the transient photocurrents when no electric field is applied to the structure (zero bias) are shown in Figure 5a,b. For a better interpretation, triangular photocurrent values were observed in both structures when the steady-state characteristics were examined, including a low illumination intensity. However, especially with the copolymer (P(EHA-*co*-AA)) structure, the fact that both the photocurrent continues to increase with time at both illumination intensities and the decay times are longer compared with the homopolymer (P(EHA)) structure can be attributed to the interface/surface state distributions (*N*_ss_) (shallow or deep traps) and/or the intensities of the trap levels that act as recombination centers in this structure.^39−42^ Later in this section, we discuss the characteristics of *N*_ss_ as a function of the difference in energy levels (*E*_c_–*E*_ss_).

**Figure 6:**
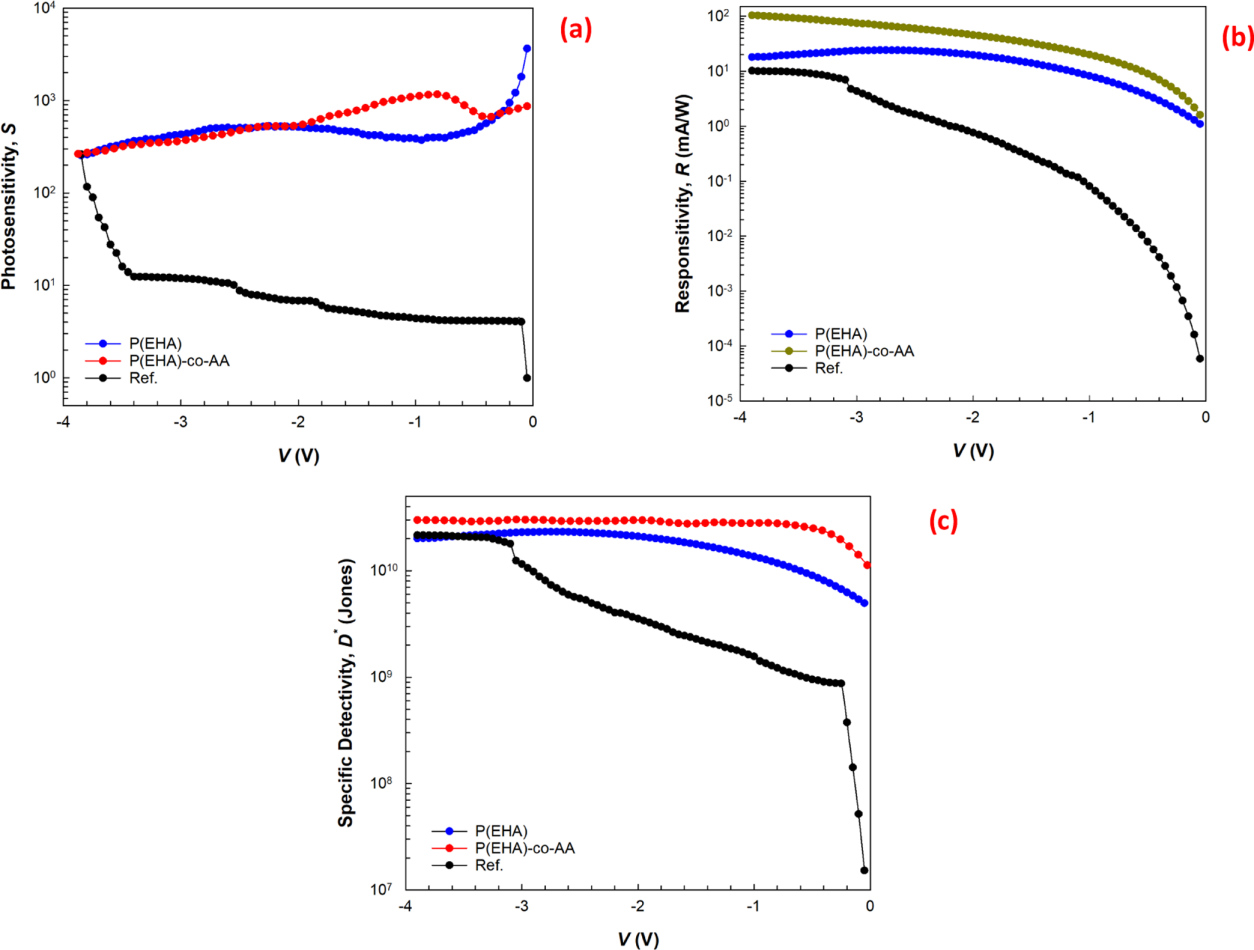
Voltage-dependent (a) photosensitivity, (b) responsitivity, and (c) specific detectivity plots of the structures.

For a better understanding of the PD characteristics under illumination, herein, PD parameters, such as *S*, *R*, and D*, were calculated using the following relationships to study the photodetector performance and plotted in Figure 6 with a reference structure for better awareness (Figure 6a–c)
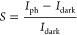
1
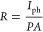
2
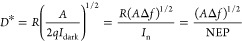
3where *I*_ph_ and *I*_dark_ are the photocurrent and dark current, *P* is denoted as the illumination intensity, *A* and *q* are denoted by the area of the diode and the charge of the electron, *I*_n_ is the noise current, Δ*f* is the noise measurement bandwidth, and NEP is the noise equivalent power. The *S*, *R*, and D* values are found to change gradually with the applied reverse potential, which is also evidence that the fabricated structures exhibit high photosensitivity in the negative/reverse bias region. From these results, it can be seen that the sensitivity of the diodes varies greatly with the bias voltage and that the P(EHA-*co*-AA) interlayer is more sensitive to illumination. However, it can be said that the differences, such as fluctuations and peaks that appear in the voltage-dependent changes, have their source in the intensity distribution of the trap levels (*N*_ss_) at the interface of the structures. In addition, the decrease in *S* values with increasing reverse bias is associated with increasing dark current (*I*_dark_) and the decrease in *R* and D* values around zero bias is clearly associated with decreasing photocurrent (*I*_ph_).

With these evaluations, it can be said that these diode structures obtained by creating separate monolayer interfaces can be used as effective photodetectors as a result of comparison with similar literature studies.^43−46^

Meanwhile, other parameters to study the photovoltaic performance of the constructed heterostructures, the open-circuit voltage (*V*_oc_), short-circuit current (*I*_sc_), and fill factor (FF) were evaluated by using the illuminated *I*–*V* curve. The FF was calculated using the following equation^47^
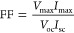
4

Here, *V*_max_ and *I*_max_ are the maximum voltage and maximum current and *I*_sc_ and *V*_oc_ denote the short-circuit photocurrent and open-circuit voltage values acquired using the *y*-axis and *x*-axis intersecting points of the illuminated *I*–*V* curve, respectively. The results of the photovoltaic parameters of the structures are listed in Table 1.

**Table 1:** Some Photovoltaic Parameters of the Structures

interlaid with	*V*_max_ (V)	*I*_max_ (A)	*V*_oc_ (V)	*I*_sc_ (A)	FF (%)
P(EHA)	0.11	2.81 × 10^–7^	0.50	6.58 × 10^–7^	9
P(EHA-*co*-AA)	0.10	1.53 × 10^–6^	0.18	2.81 × 10^–6^	30

Low FF values have been attained as a result of the obstructive impact caused by the series resistance and the interface’s susceptibility to illumination conditions. Furthermore, these interface states may have acted as sources of leakage currents and as traps for carriers generated by illumination. However, these values are sufficient for applications in photodetector/diode devices, and the affordability and simplicity of manufacturing organic materials enable their practical utilization in commercial applications.^47^

The *I*–*V* characteristics of the structures under forward bias at moderate voltage levels (*V* ≥ 3*kT*/*q*) can be analyzed by employing the thermionic emission (TE) theory. According to this theory, the equation is as follows^48,49^
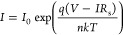
5

Here, the value of *I*_0_, which represents the saturation current at zero bias voltage in reverse bias, can be acquired by locating the point of intersection between the linear ln *I–V* fit plot and zero bias. Employing the *I*_0_ values, zero-bias barrier height (Φ_B_) values are obtained with the following equation
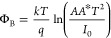
6

In eq 6, A* is the effective Richardson constant (112 A cm^–2^ K^–2^), *T* is the room temperature in kelvin, and *k* is the Boltzmann constant. Ideality factor (*n*) obtained from the slope equation of the linear fit plot of ln *I*–*V* and the corresponding equation is as follows
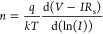
7

To compare the rectifier behavior (RR = *I*_F_/*I*_R_), *n, I*_0_, and Φ_B_ of the structures, semilogarithmic *I–V* characteristics are shown in Figure 4a,b for the dark and 100 mW cm^–2^, respectively. The experimental values of them were calculated using eqs 5–7 in the dark and under illumination and are also given in Table 2. It is seen that the photodiode with the P(EHA-*co*-AA) interlayer has a higher RR value compared to the photodiode with the P(EHA) interlayer. In other words, Table 2 clearly shows that the structure interlaid with P(EHA-*co*-AA) enables a significant reduction in leakage current compared to the structure interlaid with P(EHA). In Table 2, their ideality factors are greater than the ideal case (*n* = 1). In an ideal diode, the ideality factor is equal to 1. However, in practice, the ideality factor is often greater than 1 for semiconductor diodes. The ideality factor for a semiconductor diode is affected by a combination of factors, including nonideal current flow, formation of barrier inhomogeneities, generation–recombination effects, series resistance, the spatial intensity distribution of *N*_ss_, and cleaning of surface processes.^50^ These factors can cause deviations from the ideal behavior described by the ideal diode equation and result in an ideality factor greater than 1.^48,49^

**Table 2:** Some of the Main Electric Parameters of the Structures in the Dark and under 100 mW cm^–2^ Illumination

	dark								under 100 mW cm^–2^						
	TE				Cheung’s				TE			Cheung’s			
interlaid with	RR	*I*_0_ (pA)	*n*	Φ_B_ (eV)	*n* (d*V*/dln *I*)	Φ_B_ (eV) (*H*(*I*))	*R*_s_ (Ω) (*H*(*I*))	*R*_s_ (Ω) (d*V*/dln *I*)	*I*_0_ (nA)	*n*	Φ_B_ (eV)	Φ_B_ (eV) (*H*(*I*))	*n* (d*V*/dln* I*)	*R*_s_ (kΩ) (*H*(*I*))	*R*_s_ (kΩ) (d*V*/dln* I*)
P(EHA)	2.70 × 10^2^	8.3	4.15	0.92	4.41	0.97	1.63 × 10^5^	2.08 × 10^5^	0.42	4.60	0.81	0.77	5.58	6.16	7.21
P(EHA-*co*-AA)	1.83 × 10^3^	97.1	7.09	0.88	4.59	0.92	1.37 × 10^5^	2.44 × 10^5^	268	6.99	0.68	0.68	5.08	4.61	5.41

**Figure 7:**
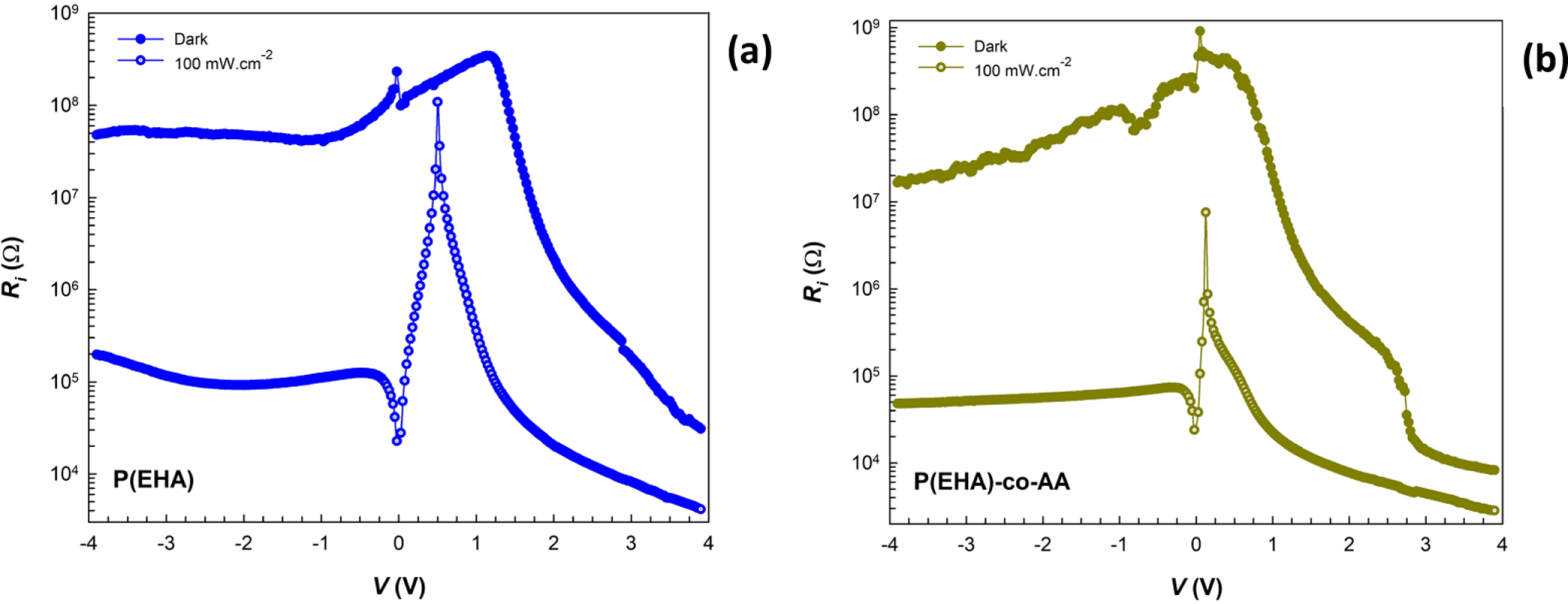
*R*_*i*_ vs *V* plots of the structures interlaid with (a) P(EHA) and (b) P(EHA-*co*-AA) in the dark and under illumination.

Both series (*R*_s_) and shunt (*R*_sh_) resistances are important parameters that affect the performance of PDs. While *R*_s_ affects the linearity, speed, and responsivity of the photodiode, *R*_sh_ affects the dark current and noise characteristics of the photodiode. Thus, both the *R*_s_ and *R*_sh_ values were obtained from the plot of the structure resistance (*R*_*i*_) versus applied bias voltage (*V*_*i*_) in the dark and under illumination. Here, *R*_*i*_*=* d*V*_*i*_/d*I*_*i*_ values are plotted, as shown in Figure 7a,b. The *R*_s_ and *R*_sh_ values obtained correspond to enough high forward/reverse bias as 1.84 × 10^5^ and 4.89 × 10^7^ Ω in the dark and 8.22 × 10^3^ and 9.64 × 10^4^ Ω under illumination for interlaid with the P(EHA) structure and 1.37 × 10^4^ and 3.66 × 10^7^ Ω in the dark and 4.47 × 10^3^ and 5.34 × 10^4^ Ω under illumination for interlaid with the P(EHA-*co*-AA) structure, respectively.

The linear part of the ln* I*–*V* plots is noticeably smaller for forward bias when *R*_s_ is very high, and in this situation, the accuracy of extracting the electrical characteristics with the *I–V* data is called into question. However, Cheung’s functions^51^ can be employed as an alternative technique, taking advantage of the downward curvature of the *I*–*V* curves of the forward bias voltage to determine the *n*, *R*_s_, and Φ_B_ quantities in the dark and under illumination. The functions of Cheung are given as follows
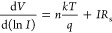
8
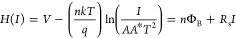
9

**Figure 8:**
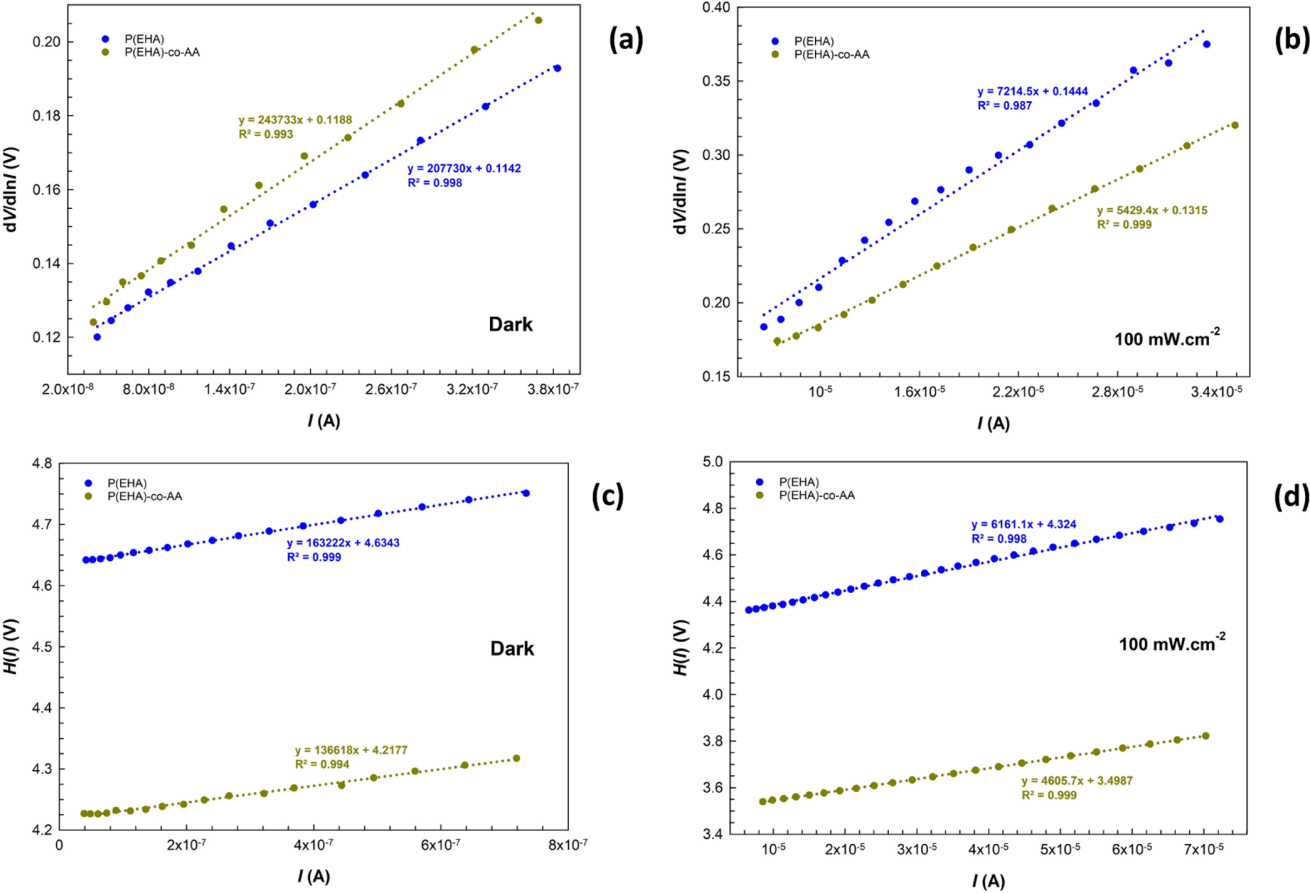
d*V*/dln *I* vs *I* plots of the structures (a) in the dark and (b) under illumination and the *H*(*I*) vs *I* plots of the structures (c) in the dark and (d) under illumination.

The plots obtained from these functions described above are shown in Figure 8a–d for both conditions. The values of *R*_s_, *n*, and Φ_B_ were calculated using the slope and intercept of d*V*/dln *I* and *H*(*I*) vs *I* plots according to eqs 8 and 9. Obtained results are also given in Table 2. When the basic parameters obtained from both approaches are examined, the increase in *n* and *I*_0_ values and the decrease in *R*_s_ and Φ_B_ values clearly indicate that the incident light energy generates excited electrons in the structures and thus a photocurrent. Particularly, high *R*_s_ values obtained in the dark have been observed in similar studies.^52^

Another critical aspect of the device that requires careful consideration is the presence of surface states/trap levels (*N*_ss_) or defects that can appear at the interface of the semiconductor and introduce energetic states or recombination centers within the band gap (*E*_g_) of the semiconductor. The characteristics of the surface traps vary based on several factors, including the chemical makeup of the interface, defects such as oxygen vacancies, and hanging boundaries at the interface.^48,53,54^ Because the sources from which they originate are different and, therefore, can be localized at different energy levels, striking effects can occur in the measured properties of the structures relative to external variables, such as voltage, temperature, frequency, and irradiation.^55−58^ The operation of the device, both in the dark and under illumination, can be significantly affected by these traps or dislocations. Using the Card and Rhoderick equations^53^ below, the forward bias *I–V* data can be employed to derive the energy density distribution profiles of *N*_ss_
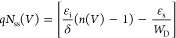
10
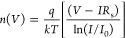
11
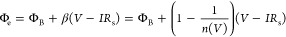
12

In these equations, *n*(*V*), Φ_e_, δ, and *W*_D_ denote the ideality factor (voltage-dependent), effective BH, interfacial layer thickness, and depletion layer width, respectively. In addition, ε_i_ and ε_s_ are the interfacial layer permittivities and the semiconductor permittivity. *W*_D_ was also calculated from the *C*–*V* measurements at 0.5 MHz. The surface state energy level (*E*_ss_) in relation to the conductance band (*E*_c_) can be represented as follows for n-type semiconductors:
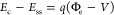
13

**Figure 9:**
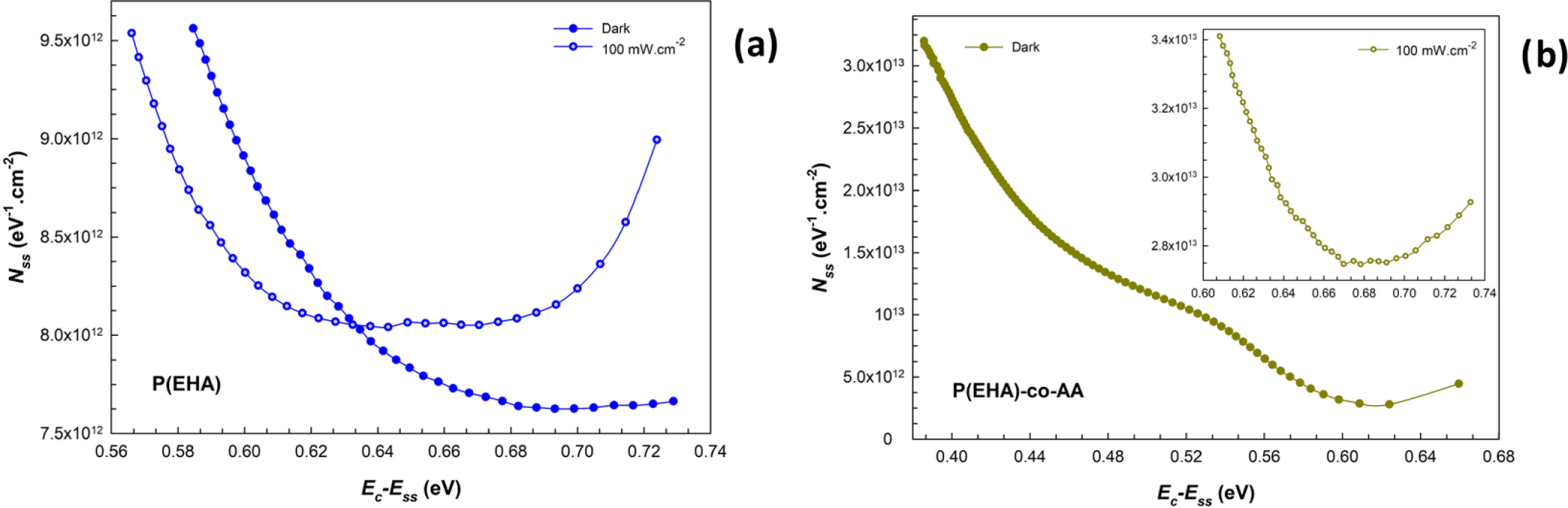
*N*_ss_ vs *E*_c_*–E*_ss_ plots of the structures interlaid with (a) P(EHA) and (b) P(EHA-*co*-AA) in the dark and under illumination.

The results are presented in Figure 9a,b to show the effects of the P(EHA) and P(EHA-*co*-AA) interlayers and the illumination on the surface states. As shown in these figures, it can be noticed that the *N*_ss_ values for both structures gradually decrease as the energy gap difference increases in the dark, but a faster increase in the *N*_ss_ values occurs for the copolymer interface structure toward the depths of the forbidden energy gap.

At these levels, called deep trap levels, the recombination centers induced by the illumination effect cause serious increases in the *N*_ss_ values. Therefore, they significantly affect the photocurrent and photodetector characteristics at certain voltages and under illumination.^59−62^ In addition, we can agree that the presence of a second molecule, combined with its molecular structure and production processes, causes the *N*_ss_ values of the copolymer interface structure to be higher than those of the homopolymer interface structure.

**Figure 10:**
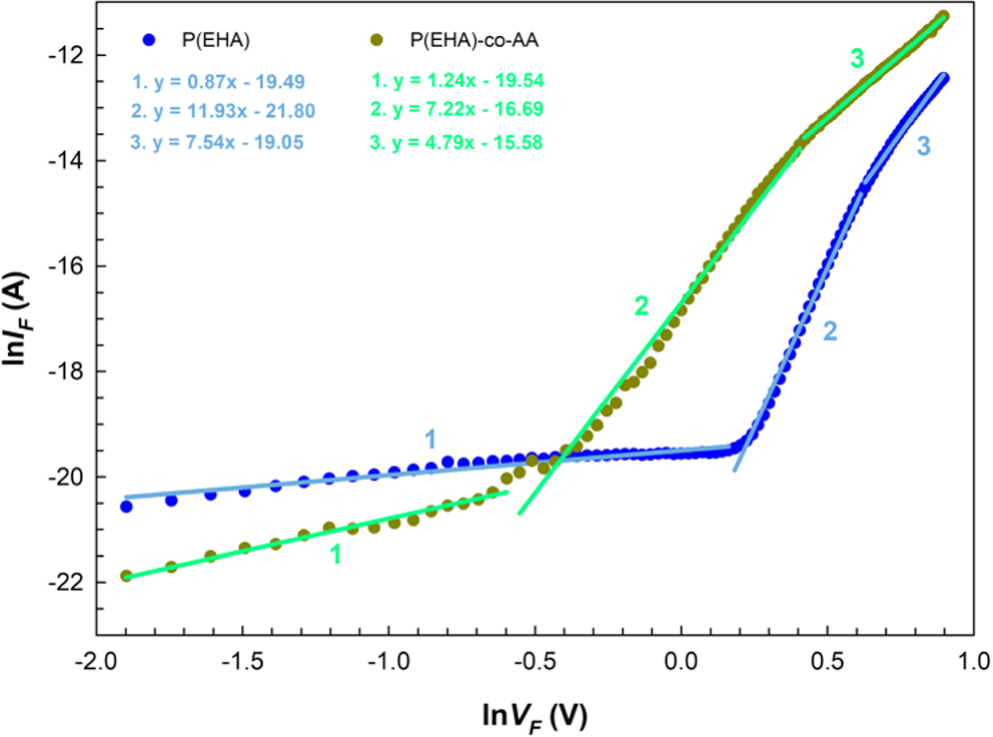
ln *I*_F_ vs ln *V*_F_ plots and linear part fit slopes of the structures in the dark.

Finally, in this section, the *I*–*V* characteristics were also constructed as double logarithmic curves and are shown in Figure 10 to identify the predominant mechanism of current conduction throughout the forward bias range of both structures in the dark.

Figure 10 illustrates that in each plot of the natural logarithm of forward current (ln* I*_F_) against the natural logarithm of forward bias (ln* V*_F_), there are three distinct linear sections with varying slopes. This relationship demonstrates that the current through the device is directly proportional to the bias voltage applied to it, indicating *I* α *V*^*m*^ (current proportional to voltage) behavior. Here, the *m* values found from the slopes of these linear regions are 0.87, 11.93, and 7.54 for interlaid with the P(EHA) structure and 1.24, 7.22, and 4.79 for interlaid with P(EHA-*co*-AA) structure. In the low bias region, the value of the exponent “*m*” is approximately 1, indicating that current conduction has an ohmic mechanism. This mechanism can be explained by the dominance of the current generated within the film itself (bulk-generated current) over the current generated by the injected free carriers. In the intermediate bias region, the primary current-limited mechanism (CLM) was identified as the trap-charge-limited current (TCLC). The value of *m* in this region is significantly greater than 2. As the electron injection increases, the traps and states within the system become filled, resulting in an accumulation of space charges throughout the process. In the high-bias region, the structure approaches the trap-filled limit; consequently, the slope tends to decrease. The intense electron injection causes electrons to escape from the traps, thereby contributing to the generation of space-charge-limited current (SCLC).^63−65^

### 3.4 *C–V* and *G*/ω*–V* Characteristics

**Figure 11:**
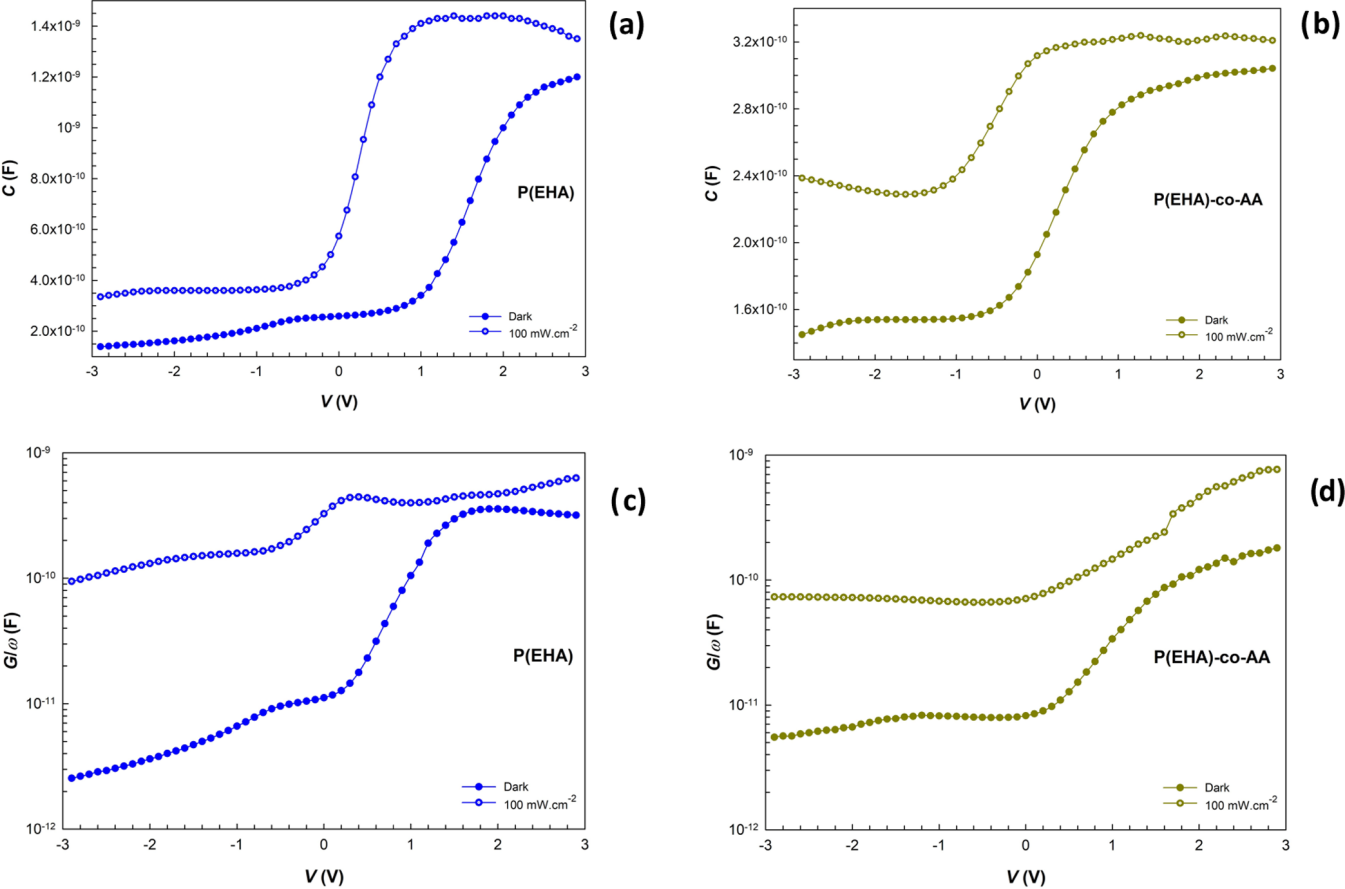
*C* vs *V* plots of the structures interlaid with (a) P(EHA), (b) P(EHA-*co*-AA), and the *G*/ω vs *V* plots of the structures interlaid with (c) P(EHA) and (d) P(EHA-*co*-AA) in the dark and under illumination.

The *C–V* and *G*/ω*–V* characteristics of the structures were measured at room temperature and 0.5 MHz. The measurements were executed under the same conditions as for the *I*–*V* measurements. The comparisons of these characteristics were made for both reverse and forward bias voltages. The results of these measurements are plotted in Figure 11a–d.

Since the interface states can easily keep up with the signal changes at low frequencies, the illumination effects are examined at a higher frequency value (0.5 MHz) to eliminate the noise effects. Particularly, the alterations in *C* and *G*/ω values are functions of illumination and voltage in the depletion regions due to the peculiar intensity distribution of *N*_ss_ at the interlayer/n-Si interface and the generation of e^–^–h^+^ pairs under the illumination effect. In addition, as seen in Figure 11a,b, the tendency of *C* values to increase in the copolymer interface structure at higher reverse biases in the inversion region can be addressed by the localized surface/interface states or trap levels in this region. This situation can also be related to the noise effect in the leakage current in the ln *I*–*V* plot (see Figure 4b).^66^

When light illuminates the device, additional e^–^–h^+^ pairs are generated through optical absorption. The mechanism behind this effect is known as the photovoltaic effect or photoconductive effect. If the energy of the photons exceeds the energy gap (*E*_g_) of the semiconductor, the emission of e^–^–h^+^ pairs in the depletion region of the semiconductor is possible. These e^–^–h^+^ pairs would then be separated at the grain boundaries by a strong local internal electric field when the structures are subjected to an electric field. These results confirm that the devices, especially the structure interlaid with P(EHA), exhibit photocapacitive behavior, which is related to the photogenerated charges. The charge carriers are generated under illumination and accumulated at the interface. As a result, the diode exhibits additional photocapacitance and conductance.

The profile of voltage-dependent *N*_ss_ was also determined by analyzing the capacitance data obtained in the dark and under illuminations, using the following equation^54,67^
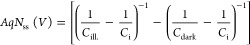
14

In the above equation, *C*_dark_ denotes the capacitance value measured in the dark, *C*_ill._ denotes the capacitance value measured under illumination, and *A* denotes the area of the rectifier contacts. In this method, which basically allows the examination of *N*_ss_ changes based on voltage by low-high frequency measurements, the *C* value measured under illumination corresponds to the low frequency (*C*_lf_ → *C*_ill._) and the *C* value measured in the dark corresponds to the high frequency (*C*_hf_ → *C*_dark_). Using this approach, which allows very practical calculation over the area between these two plots of measurements, the *N*_ss_ values are determined by isolating and separating its capacitance contribution from the observed *C–V* curve. In the equivalent circuit of Schottyk structures, the insulator capacitance (*C*_i_) is connected in series with the parallel combination of the capacitance associated with the interface/surface states or trap level capacitance (*C*_it_) and the capacitance related to space charge (*C*_sc_). Typically, in the dark and at high frequencies, these states or levels with very short relaxation times compared with the signal polarization time cannot sufficiently keep up with the ac excitation. As a result, they are not able to contribute directly to the total capacitance and conductance of the structure.^68^

**Figure 12:**
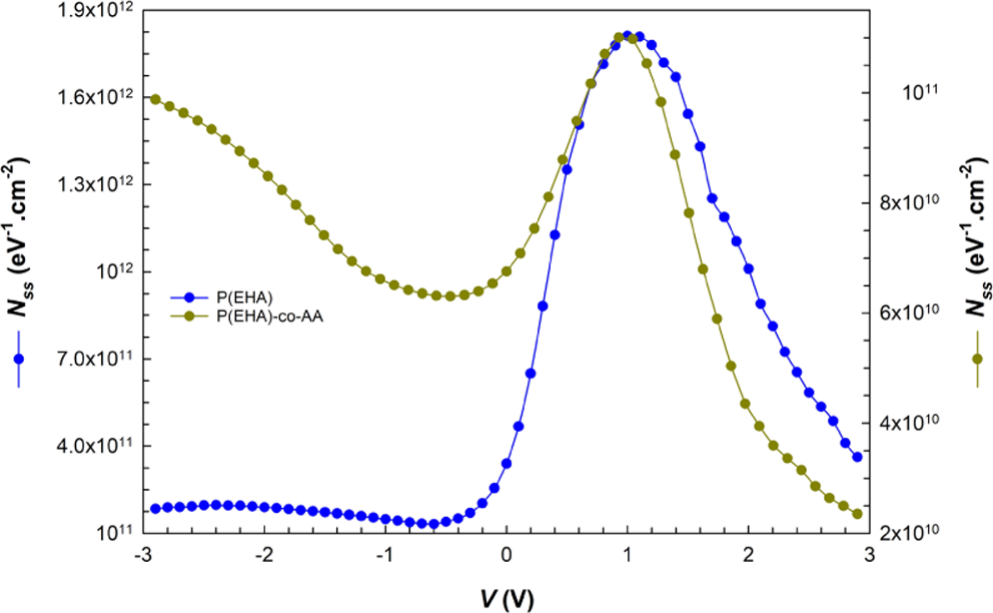
*N*_ss_ vs *V* plots of the structures obtained from *C*–*V* measurements.

The intensity distributions of *N*_ss_ as a function of the applied bias voltage are shown in Figure 12. The plots show a peak that occurs at approximately 1 V. We can say that the peculiar distribution of the *N*_ss_ is concentrated at this voltage, which coincides with the depletion region, and the differences in the order of *N*_ss_ values compared to the *I*–*V* data are due to the differences in the methods used to obtain these results. The interface states between the semiconductor and the polymer interfacial layer can be credited with similar results.^69,70^

It should also be noted that the *C* and *G*/ω values for both structures are anchored at higher forward biases due to the predominance of *R*_s_. The voltage-dependent profiles of *R*_s_ for the structures were derived from the measured *C* and *G* values (*C*_m_ and *G*_m_) in the dark and under illumination employing the equation as follows^54^
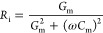
15

Here, ω represents the angular frequency (equal to 2π*f*). It is important to emphasize that the actual value of *R*_s_ corresponds to the measurements obtained with *C*_ma_ and *G*_ma_, which represent the measured capacitance and conductance values at the strong accumulation region.

**Figure 13:**
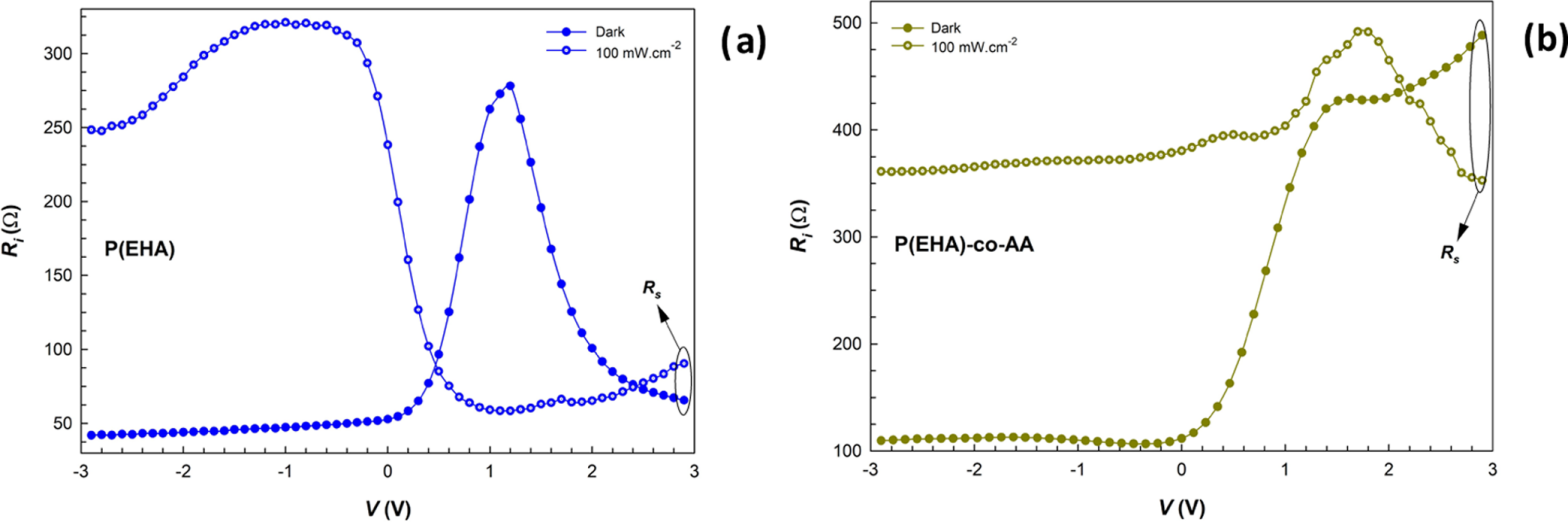
*R*_i_ vs *V* plots of the structures interlaid with (a) P(EHA) and (b) P(EHA-*co*-AA) in the dark and under illumination.

The predicted *R*_s_ values are marked in Figure 13a,b. The different characteristics of both *R*_s_ and *R*_i_ as a function of voltage and illumination effect, e.g., the peaks occurring in different voltage ranges, are a result of the nature and relaxation times of the interface/surface states discussed in detail so far in this part of the study and their reorientation and realignment by the electric field effect.^71−73^

## 4 Conclusions

In the present work, both poly(2-ethylhexyl acrylate) (PEHA) homopolymer and its copolymer combined with acrylic acid P(EHA-*co*-AA) were employed as an interface on n-Si by the iCVD method. Compared to solution-phase methods, this method offers some important advantages such as much better deposition control and homogeneous coating. After the fabrication of the device, the photodiode, photodetector, and photocapacitor characteristics of these structures were investigated in the dark and under illumination at 100 mW cm^–2^. Some of the photodetector parameters (*R*, *S*, and D*) and photoresponse characteristics of the structures showed alterations that were very sensitive to illumination and voltage. In addition, the notable changes in the values of the basic diode parameters (*n*, *I*_0_, Φ_B_, *R*_s_, and *R*_sh_) obtained with different approaches strongly indicate that the fabricated structures have photodetector and photodiode characteristics. It is clearly observed that structures with similar sensitivity in the *C*–*V* measurements exhibit photocapacitor behavior. All of these properties are also related to the interface/surface states, and their importance in the characterization of such surface- and interface-based devices is discussed in detail. The fact that the photodetector properties of the copolymer interface structure are better than the other structure can be addressed to the fact that this interface is more sensitive to light, as well as to the interface/surface states or trap level intensities. Acting as recombination centers in the forbidden band gap, these levels lead to additional increases in photocurrent under illumination. The experimental findings demonstrate that the optoelectrical properties of the structures, which have not been extensively studied, are highly sensitive to both illumination and applied bias voltage. Such functional polymers, which are in many ways more attractive to researchers than inorganic interface applications, are more likely to find their place in flexible optoelectronic devices and sensor applications. For this reason, as in this study, more detailed studies and research are crucial for today’s autonomous technology that makes our lives easier.

## References

[ref1] Tomozawa H.; Braun D.; Phillips S. D.; Worland R.; Heeger A. J.; Kroemer H. Metal-Polymer Schottky Barriers on Processible Polymers. Synth. Met. 1989, 28 (1–2), 687–690. 10.1016/0379-6779(89)90591-2.

[ref2] Yang D.; Ma D. Development of Organic Semiconductor Photodetectors: From Mechanism to Applications. Adv. Opt Mater. 2019, 7 (1), 1800522 10.1002/adom.201800522.

[ref3] Ying S.; Ma Z.; Zhou Z.; Tao R.; Yan K.; Xin M.; Li Y.; Pan L.; Shi Y. Device Based on Polymer Schottky Junctions and Their Applications: A Review. IEEE Access 2020, 8, 189646–189660. 10.1109/ACCESS.2020.3030644.

[ref4] Yang R.; Benner M.; Guo Z.; Zhou C.; Liu J. High-Performance Flexible Schottky DC Generator via Metal/Conducting Polymer Sliding Contacts. Adv. Funct Mater. 2021, 31 (43), 2103132 10.1002/adfm.202103132.

[ref5] Meng Y.; Zhang X.; Ma Y.; Feng X. Stretchable Self-Powered Generator for Multiple Functional Detection. ACS Appl. Electron. Mater. 2020, 2 (11), 3577–3584. 10.1021/acsaelm.0c00600.

[ref6] Zhang B.; Liu J.; Ren M.; Wu C.; Moran T. J.; Zeng S.; Chavez S. E.; Hou Z.; Li Z.; LaChance A. M.; Jow T. R.; Huey B. D.; Cao Y.; Sun L. Reviving the “Schottky” Barrier for Flexible Polymer Dielectrics with a Superior 2D Nanoassembly Coating. Adv. Mater. 2021, 33 (34), 2101374 10.1002/adma.202101374. 34288156

[ref7] Wang Y.; Nasreen S.; Kamal D.; Li Z.; Wu C.; Huo J.; Chen L.; Ramprasad R.; Cao Y. Tuning Surface States of Metal/Polymer Contacts Toward Highly Insulating Polymer-Based Dielectrics. ACS Appl. Mater. Interfaces 2021, 13 (38), 46142–46150. 10.1021/acsami.1c12854. 34520160

[ref8] Meng J.; Guo Z. H.; Pan C.; Wang L.; Chang C.; Li L.; Pu X.; Wang Z. L. Flexible Textile Direct-Current Generator Based on the Tribovoltaic Effect at Dynamic Metal-Semiconducting Polymer Interfaces. ACS Energy Lett. 2021, 6 (7), 2442–2450. 10.1021/acsenergylett.1c00288.

[ref9] Puneetha P.; Mallem S. P. R.; Park S. C.; Kim S.; Heo D. H.; Kim C. M.; Shim J.; An S. J.; Lee D.-Y.; Park K.-I. Ultra-Flexible Graphene/Nylon/PDMS Coaxial Fiber-Shaped Multifunctional Sensor. Nano Res. 2023, 16, 5541 10.1007/s12274-022-5235-0.

[ref10] Czech Z.; Kowalczyk A.; Kabatc J.; Świderska J. Thermal Stability of Poly(2-Ethylhexyl Acrylates) Used as Plasticizers for Medical Application. Polym. Bull. 2013, 70 (6), 1911–1918. 10.1007/s00289-012-0887-7.

[ref11] Şakalak H.; Karaman M. All-Dry Synthesis of Poly(2-Ethylhexyl Acrylate) Nanocoatings Using Initiated Chemical Vapor Deposition Method. Prog. Org. Coat. 2019, 132, 283–287. 10.1016/j.porgcoat.2019.03.044.

[ref12] Peykova Y.; Lebedeva O. V.; Diethert A.; Müller-Buschbaum P.; Willenbacher N. Adhesive Properties of Acrylate Copolymers: Effect of the Nature of the Substrate and Copolymer Functionality. Int. J. Adhes. Adhes. 2012, 34, 107–116. 10.1016/j.ijadhadh.2011.12.001.

[ref13] Haloi D. J.; Singha N. K. Synthesis of Poly(2-Ethylhexyl Acrylate)/Clay Nanocomposite by *in Situ* Living Radical Polymerization. J. Polym. Sci. A: Polym. Chem. 2011, 49 (7), 1564–1571. 10.1002/pola.24577.

[ref14] Yılmaz K.; Şakalak H.; Gürsoy M.; Karaman M. Initiated Chemical Vapor Deposition of Poly(Ethylhexyl Acrylate) Films in a Large-Scale Batch Reactor. Ind. Eng. Chem. Res. 2019, 58 (32), 14795–14801. 10.1021/acs.iecr.9b02213.

[ref15] Zhao C.; Li L.-Y.; Guo M.-M.; Zheng J. Functional Polymer Thin Films Designed for Antifouling Materials and Biosensors. Chem. Pap. 2012, 66 (5), 323–339. 10.2478/s11696-012-0147-1.

[ref16] Lee J. H.; Myung M. H.; Baek M. J.; Kim H.-S.; Lee D. W. Effects of Monomer Functionality on Physical Properties of 2-Ethylhexyl Acrylate Based Stretchable Pressure Sensitive Adhesives. Polym. Test 2019, 76, 305–311. 10.1016/j.polymertesting.2019.03.033.

[ref17] Back J.-H.; Kwon Y.; Roldao J. C.; Yu Y.; Kim H.-J.; Gierschner J.; Lee W.; Kwon M. S. Synthesis of Solvent-Free Acrylic Pressure-Sensitive Adhesives *via* Visible-Light-Driven Photocatalytic Radical Polymerization without Additives. Green Chem. 2020, 22 (23), 8289–8297. 10.1039/D0GC02807J.

[ref18] Gupta R.; Misra S. C. K.; Malhotra B. D.; Beladakere N. N.; Chandra S. Metal/Semiconductive Polymer Schottky Device. Appl. Phys. Lett. 1991, 58 (1), 51–52. 10.1063/1.104441.

[ref19] Cárdenas J.; de Vasconcelos E. A.; de Azevedo W. M.; da Silva E. F.; Pepe I.; da Silva A. F.; Ribeiro S. S.; Silva K. A. A Conducting Polymer–Silicon Heterojunction as a New Ultraviolet Photodetector. Appl. Surf. Sci. 2008, 255 (3), 688–690. 10.1016/j.apsusc.2008.07.038.

[ref20] Hu Y.; Zhou J.; Yeh P.-H.; Li Z.; Wei T.-Y.; Wang Z. L. Supersensitive, Fast-Response Nanowire Sensors by Using Schottky Contacts. Adv. Mater. 2010, 22 (30), 3327–3332. 10.1002/adma.201000278. 20517870

[ref21] Zhou X.; Yang D.; Ma D. Extremely Low Dark Current, High Responsivity, All-Polymer Photodetectors with Spectral Response from 300 Nm to 1000 Nm. Adv. Opt Mater. 2015, 3 (11), 1570–1576. 10.1002/adom.201500224.

[ref22] García de Arquer F. P.; Armin A.; Meredith P.; Sargent E. H. Solution-Processed Semiconductors for next-Generation Photodetectors. Nat. Rev. Mater. 2017, 2 (3), 16100 10.1038/natrevmats.2016.100.

[ref23] Dhar S.; Majumder T.; Mondal S. P. Graphene Quantum Dot-Sensitized ZnO Nanorod/Polymer Schottky Junction UV Detector with Superior External Quantum Efficiency, Detectivity, and Responsivity. ACS Appl. Mater. Interfaces 2016, 8 (46), 31822–31831. 10.1021/acsami.6b09766. 27800675

[ref24] Ngo T. D.; Lee M.; Yang Z.; Ali F.; Moon I.; Yoo W. J. Control of the Schottky Barrier and Contact Resistance at Metal–WSe_2_ Interfaces by Polymeric Doping. Adv. Electron. Mater. 2020, 6 (10), 2000616 10.1002/aelm.202000616.

[ref25] Altındal Ş.; Azizian-Kalandaragh Y.; Ulusoy M.; Pirgholi-Givi G. The Illumination Effects on the Current Conduction Mechanisms of the Au/(Er_2_O_3_ PVC)/n-Si (MPS) Schottky Diodes. J. Appl. Polym. Sci. 2022, 139 (27), e52497 10.1002/app.52497.

[ref26] Mousavi S. S.; Sajad B.; Majlesara M. H. Fast Response ZnO/PVA Nanocomposite-Based Photodiodes Modified by Graphene Quantum Dots. Mater. Des 2019, 162, 249–255. 10.1016/j.matdes.2018.11.037.

[ref27] Islam A.; Li J.; Pervaiz M.; Lu Z.-H.; Sain M.; Chen L.; Ouyang X. Zwitterions for Organic/Perovskite Solar Cells, Light-Emitting Devices, and Lithium Ion Batteries: Recent Progress and Perspectives. Adv. Energy Mater. 2019, 9 (10), 1803354 10.1002/aenm.201803354.

[ref28] Elamen H.; Badali Y.; Ulusoy M.; Azizian-Kalandaragh Y.; Altındal Ş.; Güneşer M. T. The Photoresponse Behavior of a Schottky Structure with a Transition Metal Oxide-Doped Organic Polymer (RuO_2_:PVC) Interface. Polym. Bull. 2023, 1–20. 10.1007/s00289-023-04725-5.

[ref29] Son H. J.; Lu L.; Chen W.; Xu T.; Zheng T.; Carsten B.; Strzalka J.; Darling S. B.; Chen L. X.; Yu L. Synthesis and Photovoltaic Effect in Dithieno[2,3- *d* :2′,3′- *d* ′]Benzo[1,2- *b* :4,5- *b* ′]Dithiophene-Based Conjugated Polymers. Adv. Mater. 2013, 25 (6), 838–843. 10.1002/adma.201204238. 23161802

[ref30] Shastry T. A.; Balla I.; Bergeron H.; Amsterdam S. H.; Marks T. J.; Hersam M. C. Mutual Photoluminescence Quenching and Photovoltaic Effect in Large-Area Single-Layer MoS_2_ – Polymer Heterojunctions. ACS Nano 2016, 10 (11), 10573–10579. 10.1021/acsnano.6b06592. 27783505

[ref31] Shanti R.; Bella F.; Salim Y. S.; Chee S. Y.; Ramesh S.; Ramesh K. Poly(Methyl Methacrylate-Co-Butyl Acrylate-Co-Acrylic Acid): Physico-Chemical Characterization and Targeted Dye Sensitized Solar Cell Application. Mater. Des 2016, 108, 560–569. 10.1016/j.matdes.2016.07.021.

[ref32] Ashraf I. M.; El-Zahhar A. A. Studies on the Photoelectric Properties of Crosslinked-Poly(Acrylamide Co-Acrylic Acid) for Photo Detector Applications. Results Phys. 2018, 11, 842–846. 10.1016/j.rinp.2018.10.048.

[ref33] Park J. H.; Lee T.-W.; Chin B.-D.; Wang D. H.; Park O. O. Roles of Interlayers in Efficient Organic Photovoltaic Devices. Macromol. Rapid Commun. 2010, 31 (24), 2095–2108. 10.1002/marc.201000310. 21567636

[ref34] Bilkan Ç. Determination of Structural Properties of Some Important Polymers Used as Interfacial Layer in Fabrication of Schottky Barrier Diodes (SBDs). Iğdır Üniv. Fen Bilimleri Enst. Derg. 2020, 10 (1), 225–233. 10.21597/jist.615541.

[ref35] Liu P. Modifications of Carbon Nanotubes with Polymers. Eur. Polym. J. 2005, 41 (11), 2693–2703. 10.1016/j.eurpolymj.2005.05.017.

[ref36] Rose A. Concepts in Photoconductivity and Allied Problems; Interscience Publishers, New York, 1963.

[ref37] Mekki A.; Ocaya R. O.; Dere A.; Al-Ghamdi A. A.; Harrabi K.; Yakuphanoglu F. New Photodiodes Based Graphene-Organic Semiconductor Hybrid Materials. Synth. Met. 2016, 213, 47–56. 10.1016/j.synthmet.2015.12.026.

[ref38] Demirezen S.; Altındal Yerişkin S. A Detailed Comparative Study on Electrical and Photovoltaic Characteristics of Al/p-Si Photodiodes with Coumarin-Doped PVA Interfacial Layer: The Effect of Doping Concentration. Polym. Bull. 2020, 77 (1), 49–71. 10.1007/s00289-019-02704-3.

[ref39] Murphy T. E.; Moazzami K.; Phillips J. D. Trap-Related Photoconductivity in ZnO Epilayers. J. Electron. Mater. 2006, 35 (4), 543–549. 10.1007/s11664-006-0097-x.

[ref40] Elgazzar E. Improvement the Efficacy of Al/CuPc/n-Si/Al Schottky Diode Based on Strong Light Absorption and High Photocarriers Response. Mater. Res. Express 2020, 7 (9), 095102 10.1088/2053-1591/abb5ca.

[ref41] Hwang I.; McNeill C. R.; Greenham N. C. Drift-Diffusion Modeling of Photocurrent Transients in Bulk Heterojunction Solar Cells. J. Appl. Phys. 2009, 106 (9), 094506 10.1063/1.3247547.

[ref42] Ma L.; Zhang S.; Yao H.; Xu Y.; Wang J.; Zu Y.; Hou J. High-Efficiency Nonfullerene Organic Solar Cells Enabled by 1000 Nm Thick Active Layers with a Low Trap-State Density. ACS Appl. Mater. Interfaces 2020, 12 (16), 18777–18784. 10.1021/acsami.0c05172. 32233417

[ref43] Luo L.; Zou Y.; Ge C.; Zheng K.; Wang D.; Lu R.; Zhang T.; Yu Y.; Guo Z. A Surface Plasmon Enhanced Near-Infrared Nanophotodetector. Adv. Opt Mater. 2016, 4 (5), 763–771. 10.1002/adom.201500701.

[ref44] Marnadu R.; Shkir M.; Hakami J.; Ashraf I. M.; Baskaran P.; Sivaganesh D.; Chandekar K. V.; Kim W. K.; Gedi S. Significant Enhancement in Photosensitivity, Responsivity, Detectivity and Quantum Efficiency of Co_3_O_4_ Nanostructured Thin Film-Based Photodetectors through Mo Doping Developed by Spray Pyrolysis Method. Surf. Interfaces 2022, 34, 102366 10.1016/j.surfin.2022.102366.

[ref45] Ahmed F.; Datta J.; Sarkar S.; Dutta B.; Jana A. D.; Ray P. P.; Mir M. H. Water Tetramer Confinement and Photosensitive Schottky Behavior of a 2D Coordination Polymer. ChemistrySelect 2018, 3 (24), 6985–6991. 10.1002/slct.201801083.

[ref46] Çiçek O.; Karasüleymanoğlu M.; Kurnaz S.; Öztürk Ö.; Taşçı A. T. Self-Powered Visible-UV Light Photodiodes Based on ZnO Nanorods-Silicon Heterojunctions with Surface Modification and Structural Enhancement. Optik 2022, 261, 169137 10.1016/j.ijleo.2022.169137.

[ref47] Chaleawpong R.; Promros N.; Charoenyuenyao P.; Borwornpornmetee N.; Sittisart P.; Sittimart P.; Tanaka Y.; Yoshitake T. Photovoltaic, Capacitance-Voltage, Conductance-Voltage, and Electrical Impedance Characteristics of p-Type Silicon/Intrinsic-Silicon/n-Type Semiconducting Iron Disilicide Heterostructures Built via Facing Target Direct-Current Sputtering. Thin Solid Films 2020, 709, 138229 10.1016/j.tsf.2020.138229.

[ref48] Sze S. M. Physics of Semiconductor Devices, 2nd ed.; Wiley: New York, 1981.

[ref49] Rhoderick E. H.; Williams R. H. Metal-Semiconductor Contacts, 2nd ed.; Clarendon Press: Oxford, 1988.

[ref50] Çiçek O.; Tecimer H. U.; Tan S. O.; Tecimer H.; Altındal Ş.; Uslu İ. Evaluation of Electrical and Photovoltaic Behaviours as Comparative of Au/n-GaAs (MS) Diodes with and without Pure and Graphene (Gr)-Doped Polyvinyl Alcohol (PVA) Interfacial Layer under Dark and Illuminated Conditions. Composites, Part B 2016, 98, 260–268. 10.1016/j.compositesb.2016.05.042.

[ref51] Cheung S. K.; Cheung N. W. Extraction of Schottky Diode Parameters from Forward Current-Voltage Characteristics. Appl. Phys. Lett. 1986, 49 (2), 85–87. 10.1063/1.97359.

[ref52] Dutta B.; Das D.; Datta J.; Chandra A.; Jana S.; Sinha C.; Ray P. P.; Mir M. H. Synthesis of a Zn (ii)-Based 1D Zigzag Coordination Polymer for the Fabrication of Optoelectronic Devices with Remarkably High Photosensitivity. Inorg. Chem. Front 2019, 6 (5), 1245–1252. 10.1039/C9QI00162J.

[ref53] Card H. C.; Rhoderick E. H. Studies of Tunnel MOS Diodes I. Interface Effects in Silicon Schottky Diodes. J. Phys. D: Appl. Phys. 1971, 4 (10), 319 10.1088/0022-3727/4/10/319.

[ref54] Nicollian E. H.; Brews J. R. Metal Oxide Semiconductor (MOS) Physics and Technology; John Wiley & Sons: New York, 1982.

[ref55] Sze S. M.; Ng K. K. Physics of Semiconductor Devices, 3rd ed.; John Wiley & Sons, Inc.: Hoboken, NJ, USA, 2006.

[ref56] Nicollian E. H.; Brews J. R. MOS (Metal Oxide Semiconductor) Physics and Technology; John Wiley & Sons, 2002.

[ref57] Goetzberger A.; Klausmann E.; Schulz M. J. Interface States on Semiconductor/Insulator Surfaces. CRC Crit. Rev. Solid State Sci. 1976, 6 (1), 1–43. 10.1080/10408437608243548.

[ref58] Berktaş Z.; Orhan E.; Ulusoy M.; Yildiz M.; Altındal Ş. Negative Capacitance Behavior at Low Frequencies of Nitrogen-Doped Polyethylenimine-Functionalized Graphene Quantum Dots-Based Structure. ACS Appl. Electron. Mater. 2023, 5 (3), 1804–1811. 10.1021/acsaelm.3c00011.

[ref59] Kumar P.; Jain S. C.; Kumar V.; Chand S.; Tandon R. P. Effect of Illumination on the Space Charge Limited Current in Organic Bulk Heterojunction Diodes. Appl. Phys. A: Mater. Sci. Process. 2009, 94 (2), 281–286. 10.1007/s00339-008-4771-0.

[ref60] Khalili S.; Chenari H. M.; Yıldırım F.; Orhan Z.; Aydogan S. Highly Sensitive, Self-Powered Photodetector Based on Reduced Graphene Oxide- Polyvinyl Pyrrolidone Fibers (Fs)/p-Si Heterojunction. J. Alloys Compd. 2021, 889, 161647 10.1016/j.jallcom.2021.161647.

[ref61] Karataş Ş.; Yakuphanoğlu F. Effects of Illumination on Electrical Parameters of Ag/n-CdO/p-Si Diode. Mater. Chem. Phys. 2013, 138 (1), 72–77. 10.1016/j.matchemphys.2012.10.038.

[ref62] Labanti C.; Wu J.; Shin J.; Limbu S.; Yun S.; Fang F.; Park S. Y.; Heo C.-J.; Lim Y.; Choi T.; Kim H.-J.; Hong H.; Choi B.; Park K.-B.; Durrant J. R.; Kim J.-S. Light-Intensity-Dependent Photoresponse Time of Organic Photodetectors and Its Molecular Origin. Nat. Commun. 2022, 13 (1), 3745 10.1038/s41467-022-31367-4. 35768429 PMC9243077

[ref63] Demirezen S.; Al-Sehemi A. G.; Yüzer A.; Ince M.; Dere A.; Al-Ghamdi A. A.; Yakuphanoglu F. Electrical Characteristics and Photosensing Properties of Al/Symmetrical CuPc/p-Si Photodiodes. J. Mater. Sci.: Mater. Electron. 2022, 33 (26), 21011–21021. 10.1007/s10854-022-08906-2.

[ref64] Reddy V. R. Electrical Properties of Au/Polyvinylidene Fluoride/n-InP Schottky Diode with Polymer Interlayer. Thin Solid Films 2014, 556, 300–306. 10.1016/j.tsf.2014.01.036.

[ref65] Wagle S.; Shirodkar V. Space-Charge-Limited Conduction in Thin Film Al/Sb2Pb1Se7/Al Devices. Braz. J. Phys. 2000, 30 (2), 380–385. 10.1590/S0103-97332000000200019.

[ref66] Kaushik J. K.; Balakrishnan V. R.; Mongia D.; Kumar U.; Dayal S.; Panwar B. S.; Muralidharan R. Investigation of Surface Related Leakage Current in AlGaN/GaN High Electron Mobility Transistors. Thin Solid Films 2016, 612, 147–152. 10.1016/j.tsf.2016.06.003.

[ref67] Castagné R.; Vapaille A. Description of the SiO_2_—Si Interface Properties by Means of Very Low Frequency MOS Capacitance Measurements. Surf. Sci. 1971, 28 (1), 157–193. 10.1016/0039-6028(71)90092-6.

[ref68] Cicek O.; Arslan E.; Altindal S.; Badali Y.; Ozbay E. 21.2 MV/K High-Performance Ni _(50 nm)_ -Au _(100 nm)_ /Ga _2_ O _3_/*p* -Si Vertical MOS Type Diode and the Temperature Sensing Characteristics With a Novel Drive Mode. IEEE Sens J. 2022, 22 (24), 23699–23704. 10.1109/JSEN.2022.3219553.

[ref69] Demirezen S.; Altndal Ş.; Uslu I. Two Diodes Model and Illumination Effect on the Forward and Reverse Bias I-V and C-V Characteristics of Au/PVA (Bi-Doped)/n-Si Photodiode at Room Temperature. Curr. Appl. Phys. 2013, 13 (1), 53–59. 10.1016/j.cap.2012.06.009.

[ref70] Acar F. Z.; Buyukbas-Ulusan A.; Tataroglu A. Analysis of Interface States in Au/ZnO/p-InP (MOS) Structure. J. Mater. Sci.: Mater. Electron. 2018, 29 (15), 12553–12560. 10.1007/s10854-018-9371-y.

[ref71] Dhariwal S. R.; Mittal S.; Mathur R. K. Theory for Voltage Dependent Series Resistance in Silicon Solar Cells. Solid-State Electron. 1984, 27 (3), 267–273. 10.1016/0038-1101(84)90123-0.

[ref72] Chattopadhyay P.; Banerjee A. On the Voltage-dependent Series Resistance of a Planar Schottky Barrier Diode. Int. J. Electron. 2012, 99 (8), 1051–1061. 10.1080/00207217.2011.651696.

[ref73] Garland J. E.; Crain D. J.; Zheng J. P.; Sulyma C. M.; Roy D. Electro-Analytical Characterization of Photovoltaic Cells by Combining Voltammetry and Impedance Spectroscopy: Voltage Dependent Parameters of a Silicon Solar Cell under Controlled Illumination and Temperature. Energy Environ. Sci. 2011, 4 (2), 485–498. 10.1039/C0EE00307G.

